# Supplementary figures and images for: Upgrading Monocytes Therapy for Critical Limb Ischemia Patient Treatment: Pre-Clinical and GMP-Validation Aspects
Source: Int J Mol Sci. 2022 Oct 21;23(20):12669. doi: 10.3390/ijms232012669 (PMC9604444; doi:10.3390/ijms232012669)

Figure S1

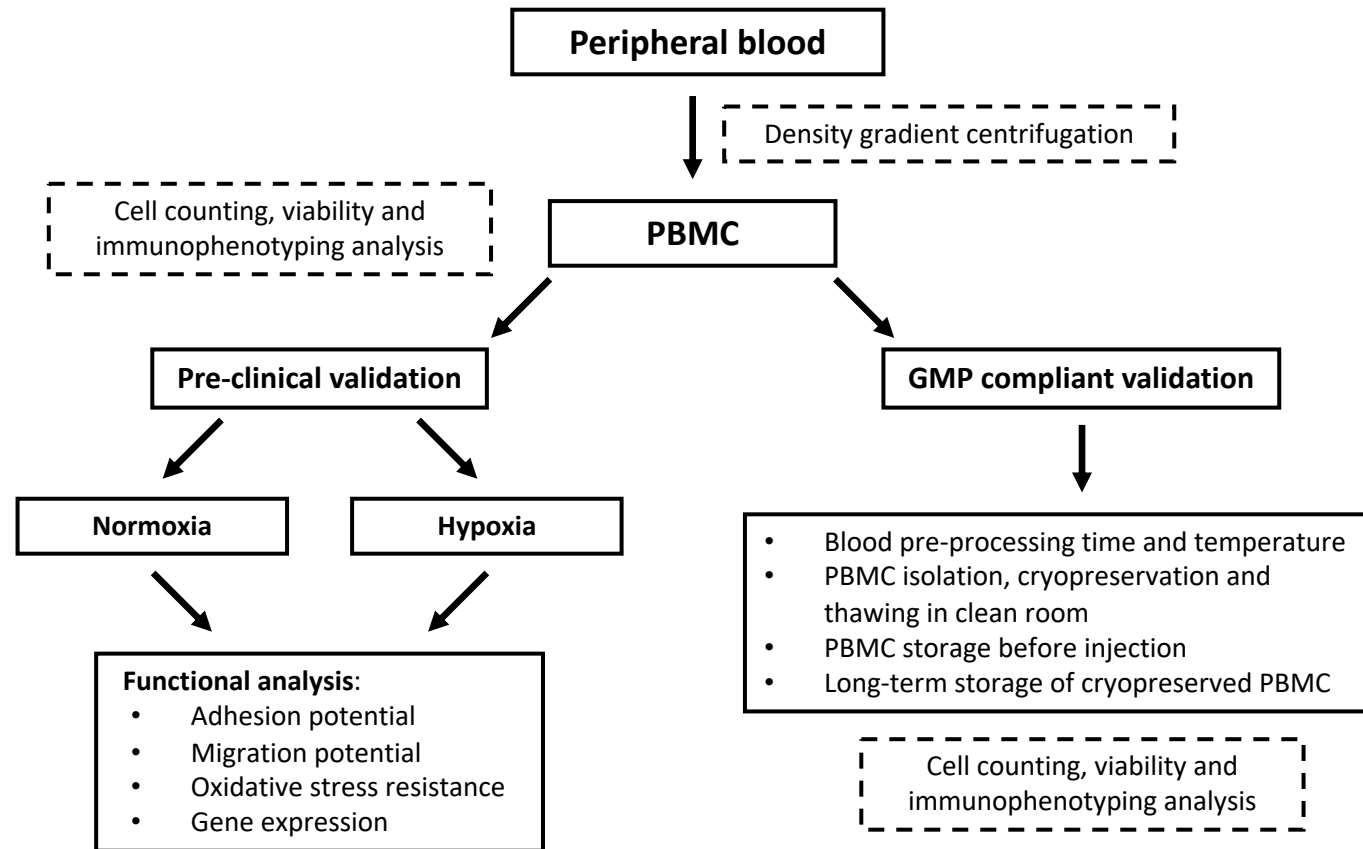

Figure S2

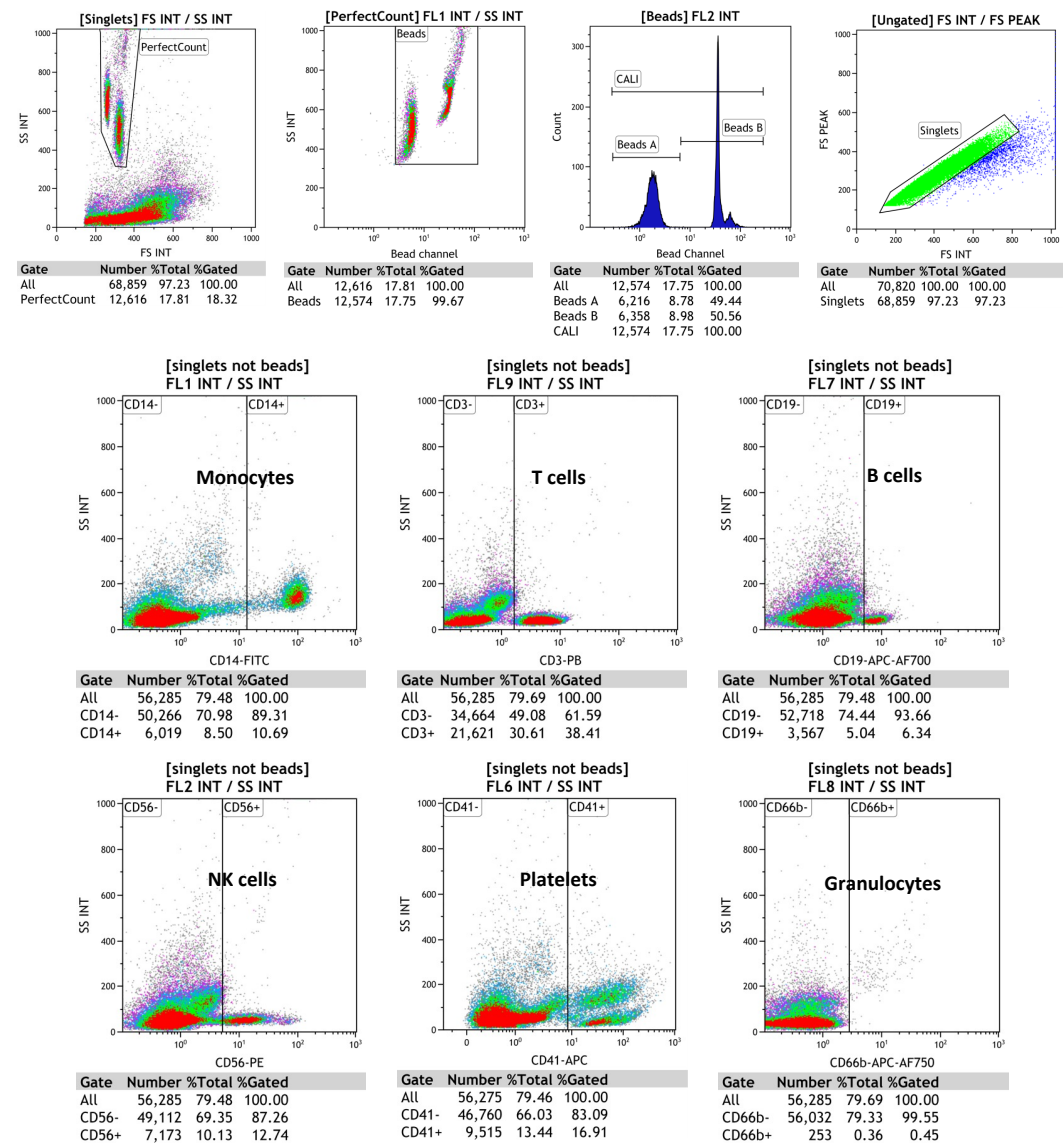

Supplement: Supplementary file 1 [file ijms-23-12669-s001.zip › ijms-1933195-supplementary.pdf]
